# Subjects with Low Plasma HDL Cholesterol Levels Are Characterized by an Inflammatory and Oxidative Phenotype

**DOI:** 10.1371/journal.pone.0078241

**Published:** 2013-11-11

**Authors:** Kirsten B. Holven, Kjetil Retterstøl, Thor Ueland, Stine M. Ulven, Marit S. Nenseter, Marit Sandvik, Ingunn Narverud, Knut E. Berge, Leiv Ose, Pål Aukrust, Bente Halvorsen

**Affiliations:** 1 Department of Nutrition, Institute for Basic Medical Sciences, University of Oslo, Oslo, Norway; 2 The Lipid Clinic, Oslo University Hospital Rikshospitalet, Oslo, Norway; 3 Research Institute of Internal Medicine, Oslo University Hospital Rikshospitalet, Oslo, Norway; 4 Department of Health, Nutrition and Management, Faculty of Health Sciences, Oslo and Akershus University College of Applied Sciences, Oslo, Norway; 5 Faculty of Medicine, University of Oslo, Oslo, Norway; 6 Medical Genetics Laboratory, Department of Genetics, Oslo University Hospital Rikshospitalet, Oslo, Norway; 7 Section of Clinical Immunology and Infectious Disease Oslo, Oslo, Norway; 8 OsloUniversity Hospital Rikshospitalet, Oslo, Norway; 9 K.G. Jebsen Inflammatory Research Center, University of Oslo, Oslo, Norway; Governmental Technical Research Centre of Finland, Finland

## Abstract

**Background:**

Epidemiological studies have shown that low plasma levels of high-density lipoprotein (HDL) cholesterol are associated with increased risk of cardiovascular disease, but the mechanisms for the possible atheroprotective effects of HDL cholesterol have still not been fully clarified, in particular in relation to clinical studies.

**Objective:**

To examine the inflammatory, anti-oxidative and metabolic phenotype of subjects with low plasma HDL cholesterol levels.

**Methods and Results:**

Fifteen subjects with low HDL cholesterol levels (eleven males and four females) and 19 subjects with high HDL (three males and 16 females) were recruited. Low HDL cholesterol was defined as ≤10th age/sex specific percentile and high HDL-C was defined as ≥90 age/sex specific percentile. Inflammatory markers in circulation and PBMC gene expression of cholesterol efflux mediators were measured. Our main findings were: (i) subjects with low plasma HDL cholesterol levels were characterized by increased plasma levels of CRP, MMP-9, neopterin, CXCL16 and ICAM-1 as well as low plasma levels of adiponectin, suggesting an inflammatory phenotype; (ii) these individuals also had reduced paraoxonase (PON)1 activity in plasma and PON2 gene expression in peripheral blood mononuclear cells (PBMC) accompanied by increased plasma levels of oxidized LDL suggesting decreased anti-oxidative capacity; and (iii) PBMC from low HDL subjects also had decreased mRNA levels of ABCA1 and ABCG1, suggesting impaired reverse cholesterol transport.

**Conclusion:**

Subjects with low plasma HDL cholesterol levels are characterized by an inflammatory and oxidative phenotype that could contribute to the increased risk of atherosclerotic disorders in these subjects with low HDL levels.

## Introduction

Epidemiological studies have shown that low plasma levels of high-density lipoprotein (HDL) cholesterol are associated with increased risk of cardiovascular disease (CVD), potentially reflecting atheroprotective effects of this lipoprotein [1]–[3]. However, low HDL cholesterol levels caused by genetic reasons have in recent studies not been associated with increased risk of myocardial infarction (MI) [4]. In addition, no clinical studies have so far shown reduced morbidity or mortality by increasing the HDL cholesterol levels as the main target [5]–[6].

The mechanisms by which HDL cholesterol could attenuate atherogenesis have not been fully elucidated, but may involve its ability to promote reverse cholesterol transport from peripheral tissue to the liver as well as its anti-inflammatory properties [7]–[10]. HDL cholesterol has been shown to protect from severe endotoxemia in both human and animal models [11]–[13], and low plasma levels of HDL cholesterol was associated with increased sensitivity toward inflammatory stimuli with subsequent enhanced inflammatory and pro-coagulant responses after endotoxin challenge in humans [13]. Of interest, inflammation has recently been suggested to negatively influence the cholesterol efflux capacity of HDL cholesterol [14] as well as changing the ability of HDL cholesterol to mediate anti-inflammatory and anti-oxidative effects, potentially reflecting a pathogenic loop between inflammation and the functional capacity of HDL cholesterol in atherogenesis.

In order to further examine the metabolic phenotype of subjects with low plasma HDL cholesterol levels, we investigated markers of inflammation and oxidative stress in subjects with very low and very high plasma levels of HDL cholesterol.

## Methods

### Subjects

Fifteen subjects with low HDL cholesterol levels (eleven males and four females) and nineteen subjects with high HDL (three males and sixteen females) were recruited in the study. Low HDL cholesterol was defined as ≤10^th^ age/sex specific percentile (HDL ≤0.9 mmol/L in men and ≤1.1 mmol/L in women) and high HDL cholesterol was defined as ≥90 age/sex specific percentile percentile (for men ≥1.7 mmol/L under 55 years, ≥1.6 mmol/L over 55 years and for women HDL-cholesterol ≥2.0 mmol/L under 35 years and 50–55 years and ≥1.9 mmol/L 35–50 years and over 60 years) as described by Nakanishi et al. [15]. Three of the subjects with low plasma HDL cholesterol had a mutation in the ATP-binding cassette (ABC) transporter A1 gene and two patients had a mutation in the apolipoprotein (apo) A1 gene, partly explaining their reduced plasma levels of HDL cholesterol. Two of the subjects in the low HDL cholesterol group and none in the high HDL cholesterol group had manifest CV disease. The subjects were recruited from the Lipid Clinic, the Medical Genetics Laboratory, Department of Genetics, Oslo University Hospital Rikshospitalet, Oslo, Oslo and Akershus University College of Applied Sciences, and among employees at the University of Oslo. The study protocol was approved by the Regional Committee of Medical Ethics and by the Norwegian Data Inspectorate. The investigation was performed according to the Declaration of Helsinki. Written informed consent was obtained from all subjects. Plasma and serum samples were collected after an overnight fast and stored at −80°C until analysis as previously described [16].

### Cell isolation

After blood collection, peripheral blood mononuclear cells (PBMC) were isolated using the BD Vacutainer Cell Preparation tubes with sodium citrate according to the manufacturer's instructions (Becton, Dickinson and Company, Franklin Lakes, NJ). Pellets were frozen and stored at −80°C prior to RNA isolation.

### Reverse transcriptase real-time quantitative polymerase chain reaction (RT-qPCR)

Total RNA was isolated from all PBMC samples using RNeasy mini kit (Qiagen, Hilden, Germany), lysis buffer with β-mercaptoethanol and RNase-Free DNase (Qiagen) and stored at −80°C. RNA quantity and quality measurements were performed using the ND 1000 Spectrophotometer (Saveen Werner, Carlson Circle Tampa, FL) and Agilent Bioanalyser (Agilent Technologies, Santa Clara, CA), respectively. All RNA samples had a RNA integrity number (RIN) >8. Four hundred ng RNA from all samples was reverse transcribed by using High Capacity RNA-to-cDNA Kit (Applied Biosystems, Foster City, CA). RT-qPCR was performed on an ABI PRISM 7900HT Sequence Detector System (Applied Biosystems) using SYBR green technologies (Sigma or Eurogentec [Seraing, Belgium]) or Custom TaqMan Array micro Fluidic cards (Applied Biosystems). Primer sequences can be provided by request. The endogenous controls glucuronidase β (GUSβ) and TATA box binding protein (TBP) were used for normalization. We use the average gene expression of two endogenous control genes when calculating the fold change in the comparative Ct method. The medians of Ct-values for the average of GUSβ and TBP were between (27.6695–27.763), with no statistical significant differences in the Ct-values for the average of GUSβ and TBP between the groups. The relative mRNA level for each transcript was calculated by the ΔΔ cycle threshold method [17].

### Cholesterol Efflux

THP-1 macrophages were lipid loaded by incubation with oxidized (ox) LDL (20 µg/ml) in growth medium (RPMI 1640 medium with 10% FCS) and 0.5 µCi/ml [H^3^]-Cholesterol (American Radiolabel Chemicals, St. Louis, MO) dissolved in ethanol. After 48 hours, radiolabelled media were removed, and the foam cells were washed twice with 0.2% BSA (w/v) in RPMI. Then serum from subjects with low or high plasma HDL (final concentration 10%), were added in RPMI 1640 medium (without FCS) and incubated for 3 hours. Thereafter, the cell medium was collected and the cells were harvested in 0.2 M NaOH. The radioactivity was measured by liquid scintillation counting using TRI-CARB 2300 TR Scintillation Counter (Packard, Meriden, CT). Data are presented as fractional (%) cholesterol efflux calculated as [dpm (media)/(dpm (media+cell-associated)]×100.

### Paraoxonase (PON)1 enzymatic activity

PON1 activity was measured in serum by EnzChek®Paraoxonase Assay Kit (Invitrogen, Eugene, OR) according to the manufacturer's instructions.

### Enzyme immunoassay (EIAs)

Serum levels of intracellular adhesion molecule (ICAM)-1, CXCL16, interleukin (IL)-8, adiponectin and matrix metalloproteinase (MMP)-9 were measured by EIAs obtained from R&D Systems (Minneapolis, MN). Serum amyloid A was measured by EIA provided by Invitrogen. Serum levels of neopterin were measured by EIA from IBL International (Hamburg, Germany). Serum levels of oxidized LDL (oxLDL) were measured by EIA provided by Mercodia (Uppsala, Sweden). The inter- and intra-assay coefficient of variation were <10% for all assays.

### Miscellaneous

Standard blood chemistry and lipid variables were measured in serum/plasma using routine laboratory methods at Oslo University Hospital. Serum level of C-reactive protein (CRP) was measured by a high-sensitivity immunoturbidimetric assay (Roche Diagnostic, Basel, Switzerland).

### Statistical Analysis

Data are given as median (min-max) if not otherwise stated. For comparisons of two groups of individuals, the Mann-Whitney *U* test or the Chi-square test for independence were used. Spearman's rank correlation coefficients were calculated to evaluate relationships between different variables. To investigate the variables most closely related to HDL cholesterol levels, a linear regression was performed for all measured biochemical variables and mRNA measurements, adjusting for variables that were imbalanced between the HDL groups (i.e. age, gender, statin use, body mass index (BMI) and triglycerides (TG)). The biochemical or mRNA variable was entered as the dependent variable, while the above mentioned variables and HDL group (categorical) was entered as independent variable by forced entry. Variables not normally distributed were log transformed prior to this analysis. Probability values (2-sided) were considered significant at values of <0.05.

## Results

### Characteristics of the participants

Compared to subjects in the high HDL cholesterol group (n = 19), subjects in the low HDL cholesterol group (n = 15) had significant higher BMI, apolipoprotein (apo) B, TG, glucose and HbA1c levels and lower levels of total cholesterol, apo A1, and free fatty acids (FFA) (Table 1). These differences were seen even if 73% of the low HDL cholesterol subjects were on current statin treatment as compared with 16% of the high HDL cholesterol subjects (*P*≤0.001). The increase level of FFA among the high cholesterol subjects is most likely due to the increased number of female subjects in the high cholesterol group [18]–[19]. There was no difference when calculating the genders separately (low compared to high HDL cholesterol men or low compared to high HDL cholesterol women in plasma FFA levels (data not shown). Two female subjects in the high cholesterol group reported use of hormone replacement therapy. From 13 of the low HDL cholesterol subjects and from 15 of the high HDL cholesterol subjects, information about the weekly alcohol intake was available. Alcohol intake was defined as “alcohol units” where one unit is equivalent to one small glass of wine or one (0.33 liter) beer or 4 ccl liqueur. Subjects with low HDL cholesterol levels (n = 13) consumed in average 4 alcohol units/week [median (0–17; min-max)] and subjects with high HDL cholesterol levels (n = 15) consumed 8 alcohol units/week [median (1–30; min-max)] (*P* = 0.061).

**Table 1:** Baseline characteristics.

	Low HDL	High HDL	*P*
	n = 15	n = 19	
**age, y**	55 (26–71)	59 (26–66)	0.498
**Female, %**	27	84	0.001
**BMI**	28 (22.6–46.1)	21.9 (17.8–24.4)**	<0.001
**Statin-users, %**	73	16	0.001
**cholesterol, mmol/l**	4.1 (2.3–7.2)	5.5 (4.5–8.2)**	0.001
**LDL, mmol/L**	2.7 (1.0–5.40)	2.8 (2.1–4.3)	0.956
**HDL, mmol/L**	0.6 (0.3–0.9)	2.6 (1.7–4.8)**	<0.001
**apoA-1, mmol/L**	1.0 (0.8–1.3)	2.2 (1.4–3.1)**	<0.001
**ApoB, mmol/L**	0.9 (0.5–1.5)	0.8 (0.6–1.8)*	0.018
**Triglycerides, mmol/L**	2.3 (0.5–16.4)	0.7 (0.3–1.4)**	<0.001
**Glucose, mmol/L**	5.7 (4.2–11.2)	5.0 (3.8–5.9)*	0.024
**Homocystein, Umol/L**	12 (7–20)	9 (5–17)	0.094
**Free fatty acids, umol/L**	0.45 (0.17–0.75)	0.56 (0.20–1.65)*	0.033
**HbA1c, %**	5.7 (5.0–7.6)	5.3 (4.9–6.0)*	0.007

Data are given as median (min-max) except when percentage is indicated.

HbA1c: n = 18 (high HDL); FFA: n = 18 (high HDL); LDL: n = 14 (low HDL).

Low and High HDL groups.

### Markers of inflammation

**Figure 1:**
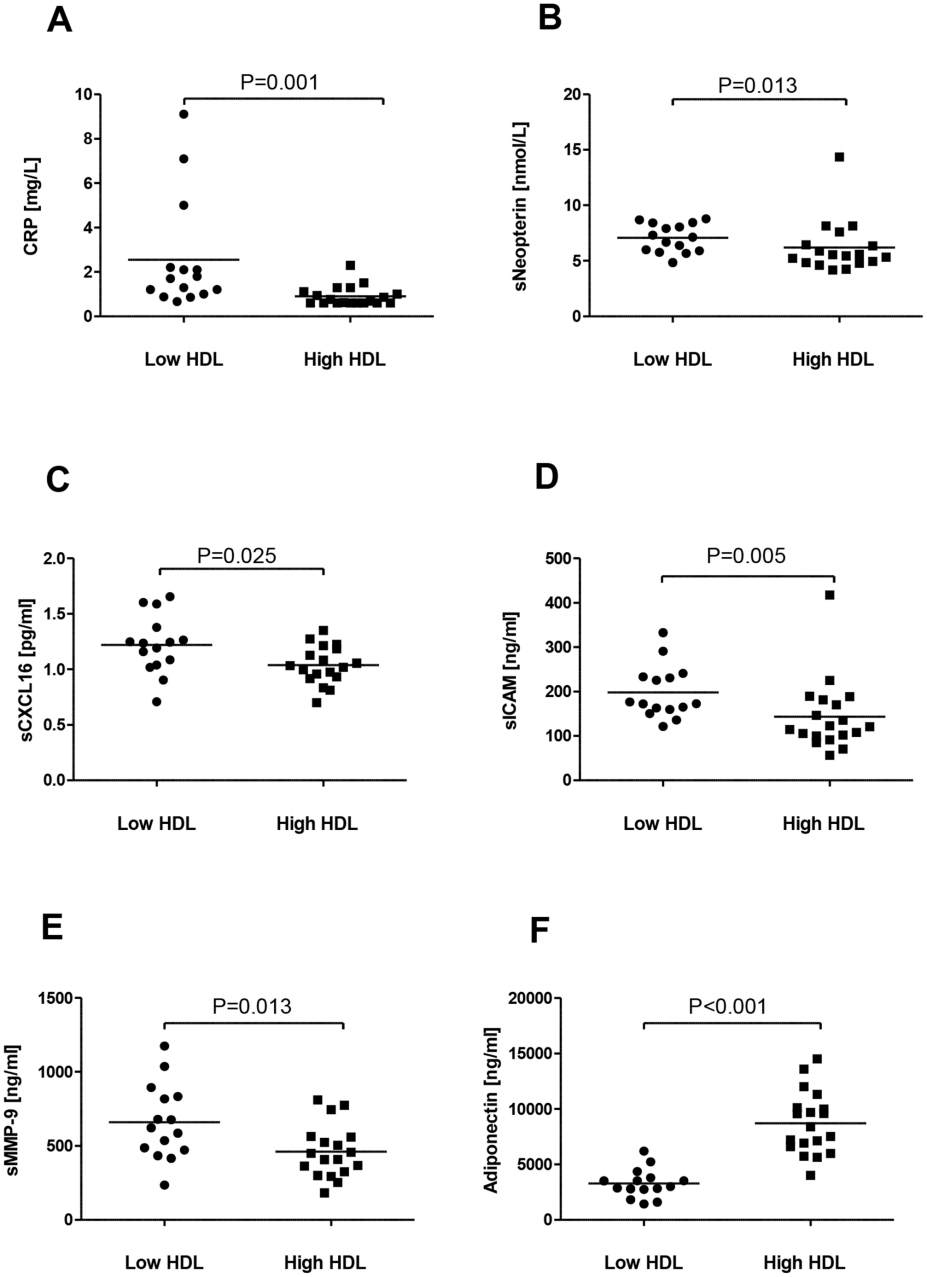
Circulating levels of inflammatory markers. CRP (A), neopterin (B), CXCL16 (C), ICAM-1 (D), MMP-9 and adiponectin (F) in subjects with low HDL cholesterol levels (n = 15) and subjects with high HDL cholesterol levels (n = 19; n = 18 for neopterin, CXCL16 and MMP-9). The horizontal bars represent median values.

Compared with those with high HDL cholesterol levels, subjects with low HDL cholesterol levels had significantly raised plasma levels of CRP, neopterin and CXCL16 (figure 1), being reliable markers of up-stream inflammatory pathways reflecting IL-6-related activity, monocyte/macrophage activation and IL-1/tumor necrosis factor (TNF)-α/interferon (IFN)γ -related activity, respectively [20]–[22]. Individuals with low HDL cholesterol levels also had higher plasma levels of ICAM-1 and MMP-9, reflecting increased leukocyte/endothelial cell interaction and matrix degrading activity, respectively (Figure 1). In contrast, individuals with low HDL cholesterol levels had decreased adiponectin levels, an adipokine with proposed anti-atherogenic, anti-inflammatory, and insulin-sensitizing effects (Figure 1). There was no difference between low HDL cholesterol statin-users (n = 11) and non-statin users (n = 3) or between high HDL cholesterol statin-users (n = 3) and non-statin users (n = 16) in the levels of CRP, neopterin or CXCL16 (data not shown). Furthermore, there was no significant difference in any of the measured inflammatory and oxidative markers between the genders in the high HDL cholesterol group. However, in the low HDL cholesterol group, neopterin was significantly lower (*P* = 0.050), and there was a trend towards significant higher levels of adiponectin (*P* = 0.068) in female subjects compared to male subjects.

### Markers of oxidative stress

**Figure 2:**
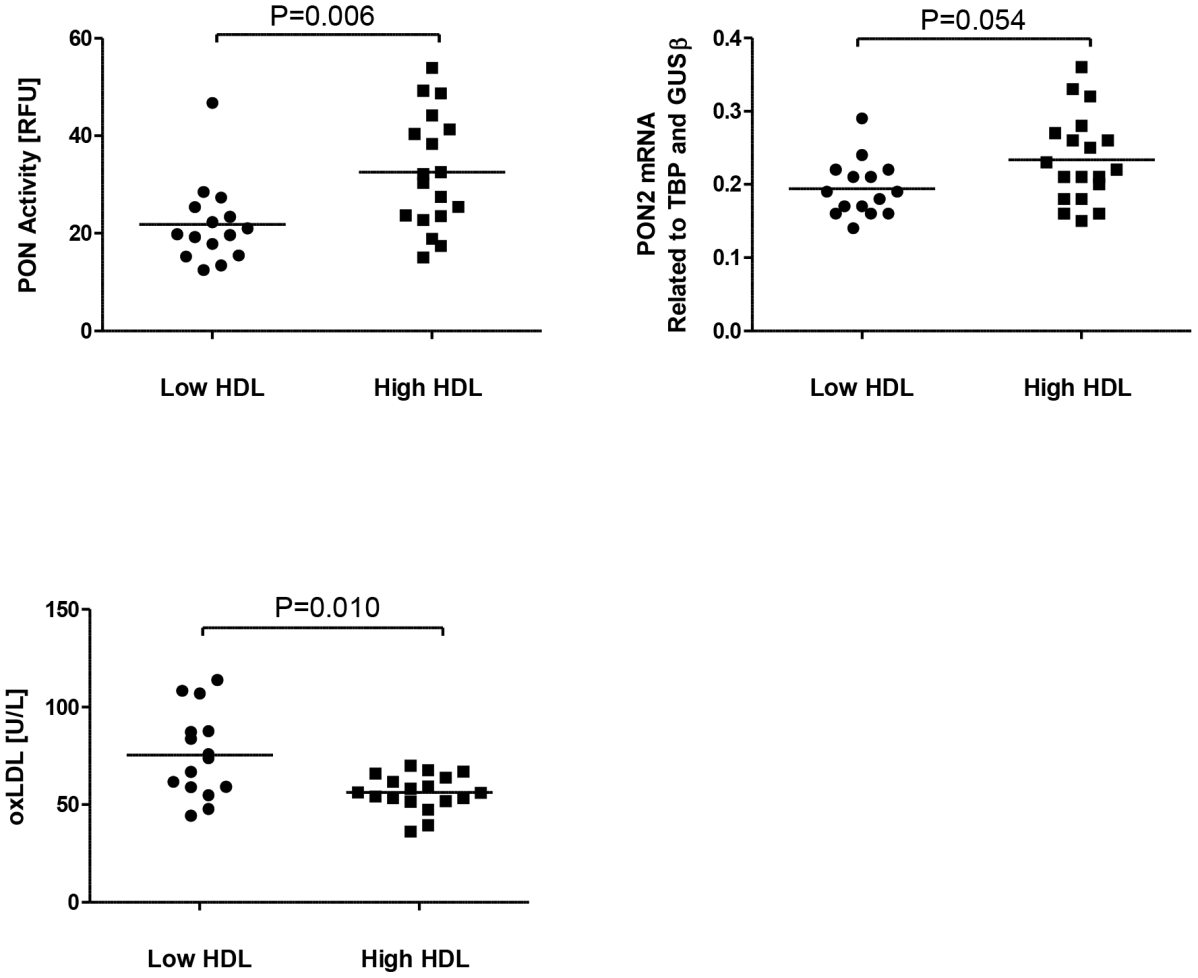
Levels of serum PON1 activity oxLDL and PBMC gene expression of PON2. Serum PON1 activity (A), PBMC gene expression levels of PON2 (B), serum oxLDL levels (C) in subjects with low HDL cholesterol levels (n = 15) and subjects with high HDL cholesterol levels (n = 19; n = 18 for PON1 activity and oxLDL). The horizontal bars represent median values.

PON has been suggested to be responsible for some of the anti-inflammatory and anti-oxidative properties of HDL [23]–[24]. Herein we found that individuals with low plasma HDL cholesterol levels had significantly lower PON1 activity in serum compared to subjects with high HDL cholesterol (Figure 2). In line with this, mRNA levels of PON2 in PBMC tended to be down-regulated in those with low HDL cholesterol levels (*P* = 0.058; Figure 2). HDL has been shown to protect LDL from oxidation [25], and indeed, we found that the circulating level of oxidized LDL (oxLDL) was significantly elevated in subjects with low HDL cholesterol (Figure 2), in spite of similar LDL-cholesterol levels in the two groups (Table 1). There was a trend towards a significant inverse correlation between plasma levels of HDL cholesterol and oxLDL (*r* = −0.311, *P* = 0.078).

### Markers of reverse cholesterol transport

**Figure 3:**
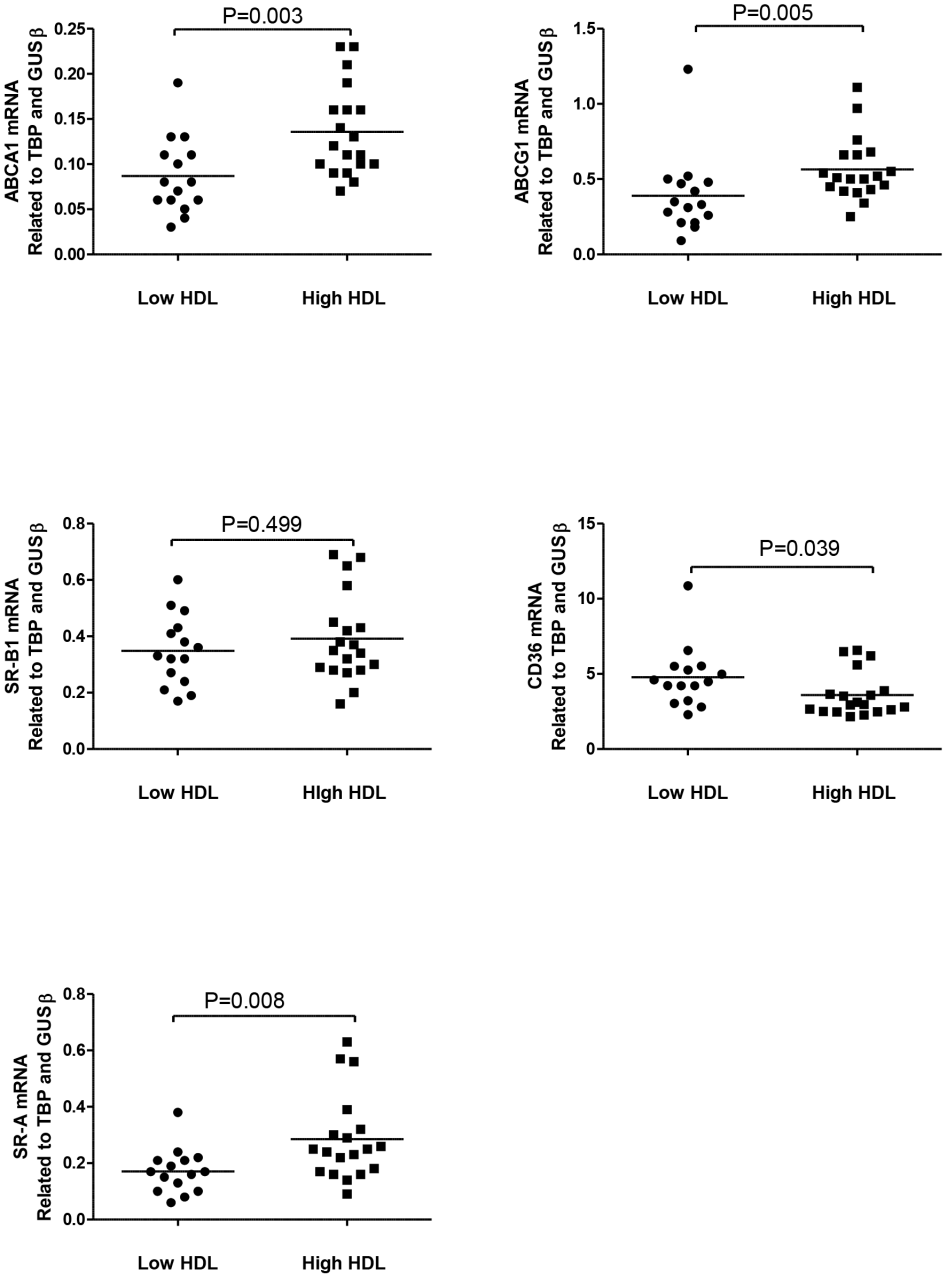
PBMC gene expression levels of cholesterol efflux mediators. ABCA1 (A), ABCG1 (B), SR-B1 (C), CD36 (D) and SR-A (E) in subjects with low HDL cholesterol levels (n = 15) and subjects with high HDL cholesterol levels (n = 19). The horizontal bars represent median values.

In the present study, we examined the ABCA1, ABCG1, SR-B1, CD36 and SR-A expression in freshly isolated PBMC. Although this is not macrophages, they may potentially be a reliable parameter of the *in vivo* situation in these patients representing a mixture of cells where monocytes are interacting with lymphocytes, exposed to an inflammatory environment which may be a mirror of the situation when monocytes are entering the vascular wall. PBMC from subjects with low HDL cholesterol levels had significantly lower mRNA levels of the ABCA1 and ABCG1, both important mediators in the reverse cholesterol transport (Figure 3). In contrast, there was no difference in mRNA levels of scavenger receptor (SR)-B1, another mediator of reverse cholesterol transport, in PBMC between the two HDL groups (Figure 3). Furthermore, whereas the mRNA expression of CD36 was significant increased, the expression of SR-A was significantly decreased in subjects with low HDL cholesterol compared to subjects with high HDL-cholesterol (*P* = 0.039 and P = 0.008; respectively) (Figure 3). While, we have no functional data on uptake of modified LDL in these cells, the result may imply that any enhanced lipid loading in monocytes from these individuals could involve CD36. There was no significant difference in any of the PBMC mRNA data between the genders in neither the low nor high HDL cholesterol group. While the expression of these transporters may seem low (The range of Ct-values for ABCA1 was 29.427–33.031 and for ABCG1 the range of Ct-values was 27.395–31.273 in the total group of participants), we find these values trustable when using TaqMan assays for quantifications.

### Cholesterol efflux capacity

We investigated the ability of serum from low HDL and high HDL cholesterol subjects to promote cholesterol efflux from lipid loaded macrophages. However, there was no difference in the cholesterol-promoting abilities of serum from low and high HDL cholesterol subjects to promote cholesterol efflux (data not shown).

### Low HDL subjects compared with subjects with normal HDL levels

In order to investigate if the low HDL cholesterol subjects differed with regard to level of inflammatory markers compared with subjects with HDL levels in the normal range, we included data from 6 subjects (4 female and 2 males) with HDL cholesterol in the normal range [1.5 (1.1–1.9) mmol/L; median (min-max)] and compared some of the parameters with the subjects with low plasma HDL cholesterol level. We found that there was no difference in mRNA expression of ABCA1 and ABCG1 between the low HDL cholesterol subjects (n = 15) and subjects with HDL cholesterol in the normal range (n = 6) (*P* = 0.139 and 0.586, respectively). However CRP and sICAM-1 was significantly higher and adiponectin levels and PON2 mRNA was significantly lower in low HDL cholesterol subjects compared to subjects with normal HDL cholesterol levels (*P* = 0.056, *P* = 0.024, *P* = 0.005 and *P* = 0.024, respectively), further supporting the notion of an unfavorably phenotype in low HDL cholesterol subjects.

### Low HDL with or without hypertriglyceridemia in general

More than 50% of the patients with low HDL cholesterol levels seem to have increased fasting TG level [26], and it has been proposed that HDL cholesterol levels may be a marker of disturbed TG metabolism. When dividing the low HDL cholesterol group into a low HDL cholesterol/low TG group (median HDL cholesterol level; 0.55 mmol/L and median TG level; 0.87 mmol/L) (n = 4) and a low HDL cholesterol/high TG group (median HDL cholesterol level: 0.83 mmol/L and median TG level: 3.86 mmol/L) (n = 11) some significant findings were revealed. The low HDL cholesterol/low TG group had significant lower plasma glucose levels (*P* = 0.013) and significant higher mRNA levels of ABCA1 (*P* = 0.025) and ABCG1 (*P* = 0.037) compared to the low HDL cholesterol/high triglyceride group, suggesting that TG levels at least in part, may influence *some* of the phenotypical characteristics of those with low HDL cholesterol levels. Interestingly, the higher mRNA level of ABCA1 in low HDL cholesterol/low TG group may suggest that the production of nascent HDL rich in apoA1 is decreased in subjects with elevated TG levels. Five of the low HDL cholesterol subjects had low HDL cholesterol levels most likely caused by mutation in the ABCA1 (n = 3) and apoA1 (n = 2) gene. There was no significant difference in any of the inflammatory markers between the low-HDL cholesterol subjects with mutation (n = 5; mutation in ABCA1 [n = 3] or apoA1 [n = 2]) compared to the other low-HDL cholesterol subjects (n = 11) with the exception of PBMC PON2 gene expression which was lower in the low HDL cholesterol subjects with mutations (*P* = 0.027). Furthermore, HbA1c (P = 0.006) and homocysteine (*P* = 0.048), which may be considered as reliable lifestyle markers was also lower in the low HDL cholesterol subjects with mutations, suggesting that the presence of low HDL cholesterol levels, independent of cause may induce a pro-inflammatory phenotype.

### Multiple Regression analysis

There were several patient characteristics that were imbalanced between patients and controls (i.e. age, gender, statin use, BMI and TG). Adjusting for these variables and HDL group by forced entry in linear regression models, showed that circulating levels of oxLDL (P = 0.051), MMP-9 (P = 0.027) and adiponectin (P = 0.022) remained significantly increased or decreased (adiponectin) in low HDL cholesterol subjects.

## Discussion

In the present study we show that subjects with low plasma HDL cholesterol levels are characterized by (i) increased levels of various inflammatory markers, (ii) decreased levels of PON activity and increased levels of oxLDL and (iii) decreased mRNA levels of mediators involved in cholesterol efflux mechanisms in macrophages. Our findings suggest that this pro-inflammatory and oxidative phenotype could contribute to an accelerated atherogenesis in these individuals.

HDL cholesterol has been shown to exert anti-inflammatory properties, at least partly by having neutralizing effects on endotoxins. In the present study we show that subjects with low HDL cholesterol levels have increased levels of several reliable markers of up-stream inflammatory pathways such as CRP, neopterin and CXCL16 as well as increased levels of ICAM-1 and MMP-9 reflecting leukocyte/endothelial interaction and matrix degradation, respectively. Importantly, this inflammatory phenotype was seen even if the use of statins, with known anti-inflammatory effects, were more abundant in the low as compared with the high HDL cholesterol group. Recently, it has been shown that inhibition of cholesterol efflux mechanisms in macrophages promotes an inflammatory phenotype in these cells [27]. The raised neopterin levels in patients with low HDL cholesterol levels, accompanied by decreased expression of ABCA1 and ABCG1 in PBMC from these subjects, may further support such a notion. In addition to raised levels of inflammatory markers, patients with low HDL levels had decreased levels of adiponectin, an adipokine with known anti-inflammatory properties, further supporting an inflammatory phenotype in relation to low HDL-levels. Interestingly, adiponectin have been shown to increase both gene expression and protein levels of ABCA1, and conversely, deficiency of adiponectin seems to suppress ABCA1 expression and ApoA-I synthesis in the liver [28]–[29], linking adiponectin to level and function of HDL cholesterol.

In addition to the increased levels of inflammatory markers, the low HDL cholesterol subjects were also characterized by reduced mRNA levels of PON2 and reduced PON1 activity in serum. PON activity *per se* has previously been shown to be inversely related to risk of CVD [30]. In addition to anti-inflammatory properties, PON activity has also been related to anti-oxidative effects of HDL. In the present study we found that subjects with low HDL cholesterol levels accompanied by decreased PON1 activity/PON2 expression also had increased oxLDL levels, despite no change in LDL levels and more abundant use of statins as compared with the high HDL cholesterol group. It is tempting to hypothesize that the high levels of oxLDL may be related to impaired anti-oxidative capacity in the low HDL group, and the inverse correlation between mRNA levels of PON2 and oxLDL may further support this notion. Moreover, as decreased anti-oxidant capacity, as shown by decreased PON level, may contribute to inflammation and *vice versa*, the combination of systemic inflammation and decreased PON activity may represent a pathogenic loop that contributes to enhanced atherogenesis in individuals with low HDL cholesterol levels. Our findings are in accordance with a recent publication from Laurila and coworkers [31], which in a large genomic and trancriptomic study showed that genetic variants within inflammatory pathways are enriched among low HDL cholesterol subjects.

Concomitantly with increased levels of inflammatory markers and oxLDL, we also found that PBMC from subjects with low HDL cholesterol levels had decreased expression of the ATP-binding cassette transporters ABCA1 and ABCG1, promoting reverse cholesterol transport [32]–[35]. Although we have no functional data in PBMC from these patients, it is not inconceivable that this profile will facilitate lipid accumulation. In contrast to our findings, Naganishi et al. [15] found no difference in the expression of ABCA1 and ABCG1 in cultured macrophages derived from subjects with low or high plasma HDL cholesterol levels, and suggested that neither the ABCA1 pathway nor the ABCG1 pathway was significantly impaired in subjects with low HDL cholesterol levels. However, in the present study we measured the expression of these genes in freshly isolated PBMC. Although this is not macrophages, they may potentially be a reliable parameter of the *in vivo* situation in these patients representing a mixture of cells where monocytes are interacting with lymphocytes, exposed to an inflammatory environment which may be a mirror of situation when monocytes are entering the vascular wall. However, as we lack protein data and functional data on cholesterol efflux in these patients our data should be interpreted with caution. In the present study there was no difference in the ability of serum derived from low HDL cholesterol or high HDL cholesterol subjects to promote efflux in lipid loaded macrophages. However, the cholesterol efflux capacity of serum may be independent of plasma HDL levels [9], [36], and further functional studies on monocytes and macrophages are needed to clarify the cholesterol efflux capacity in relation to high and low HDL cholesterol levels.

There are several limitations to the present study such as the lack of HDL subtype classification, intima-media thickness and other clinical data on the study groups. Although the use of freshly isolated PBMC has some advantages, comparative studies on monocytes-derived macrophages that also included functional assays (i.e., cholesterol efflux measurements) would clearly have strengthened our data. Furthermore, the amount of statistical analysis performed in a relatively low number of subjects and the skewed gender distribution. The strength of the study is the unique and well-characterized population with HDL levels in the extreme range (e.g. below 10 percentile and above 90 percentile) without severe hypertriglyceridemia. The finding that there was no difference in any of the inflammatory markers between the low HDL cholesterol subjects where the low HDL cholesterol was caused by a known mutation and the non-genetically low HDL cholesterol subjects supports the notion that low HDL cholesterol, independent of the cause may be associated with an inflammatory phenotype.

In conclusion, in the present study we show that subjects with low plasma HDL cholesterol levels are characterized by an inflammatory phenotype accompanied by increased levels of oxLDL and altered expression of ATP-binding cassette transporters in PBMC that could favor lipid accumulation. These characteristics, involving both the inflammatory and lipid arms of atherogenesis, could potentially contribute to increased risk of atherosclerotic disorders in subjects with low HDL cholesterol levels.
